# A case of congenital TTP presenting with microganiopathy in adulthood

**DOI:** 10.1186/2052-1839-14-16

**Published:** 2014-09-12

**Authors:** Chris D Gallivan, David M Conrad, Andrea K Kew

**Affiliations:** Department of Medicine, Division of Hematology, Division of Hematopathology, Queen Elizabeth II Health Sciences Centre, Capital District Health, Authority and Dalhousie University, Halifax, Nova Scotia Canada; Department of Pathology and Laboratory Medicine, Division of Hematopathology, Queen Elizabeth II Health Sciences Centre, Capital District Health, Authority and Dalhousie University, Halifax, Nova Scotia Canada

**Keywords:** TTP, ADAMTS13, Hemolysis, Upshaw-Schulman, Microangiopathy

## Abstract

**Background:**

Congenital thrombotic thrombocytopenic purpura (TTP), also known as Upshaw-Schulman Syndrome is a rare inherited deficiency of ADAMTS13. Unlike the more common acquired TTP which is characterized by an acquired inhibitor of ADAMTS13, patients with congenital TTP have an absolute deficiency of ADAMTS13 without an inhibitor. Congenital TTP generally presents in infancy with repeat episodes of acute hemolysis and evidence of microangiopathy, these episodes are usually triggered by illness or physiological stress. Congenital TTP can be effectively treated with plasma infusion either during acute episodes or on a prophylactic schedule to prevent episodes.

**Case presentation:**

We present a case of a 25 year old Caucasian woman with no know family history of hematological disorders with congenital TTP. She presented with episodes of hemolysis since infancy, but without clear evidence of microangiopathy until the age of 25. At presentation to our center the patient was documented to have thrombocytopenia, elevated creatinine, and schistocytes. She was initially treated with plasma infusion at a rate of 60 ml/hr continuously for a 24 hr period with resolution of her thrombocytopenia and hemolysis. At the time of writing this article she is maintained on a prophylactic schedule of biweekly plasma infusions at 10 mg/kg and is maintaining a normal platelet count with no evidence of hemolysis.

**Conclusion:**

Congenital TTP is a rare condition, and the above case is atypical as the patient did not present with clear evidence of microangiopathy until adulthood. Although this a rare condition it is important for physicians to be aware of as it, especially the possibility of atypical presentations, as the condition is potentially fatal and effective treatment exists.

## Background

Congenital thrombotic thrombocytopenic purpura (TTP), also known as Upshaw-Schulman Syndrome, is a rare inherited deficiency of A Disintegrin and Metalloproteinase with Thrombospondin Motifs 13 (ADAMTS13). In 1960 Schulman et al. described a case of an 8-year-old girl who experienced repeated episodes of thrombocytopenia that improved with plasma infusions. Schulman postulated that a factor in normal plasma that promoted platelet production or maturation was absent in his patient [1]. In 1978 Upshaw described a similar case of a 29-year-old with recurrent episodes of thrombocytopenia associated with microangiopathic hemolytic anemia (MAHA) that also responded to plasma infusions. Upshaw documented 32 episodes of MAHA in his patient over an 11-year period, the majority of which were preceded by infections or other stressors, such as pregnancy and surgery [2].

In 1982 Moake et al. identified unusually large von Willebrand Factor (vWF) multimers in the plasma of four patients with chronic relapsing TTP. He postulated that these patients lacked a plasma factor necessary for cleaving large vWF multimers [3]. It has subsequently been demonstrated that ADAMTS13 is the vWF-cleaving protease that is deficient in congenital TTP [4, 5]. Lotta et al. found that residual plasma ADAMST13 activity levels in patients with congenital TTP correlated with disease severity, as lower activity was associated with earlier onset of symptoms, requirement of plasma infusions at an earlier age, increased annual rates of episodes of TTP, and need for prophylactic plasma infusions [6]. The natural history of congenital TTP is one of relapsing episodes of TTP [7]. In a case series of 43 patients Fujimura et al. described the majority of patients, 23 out of 43 receiving an appropriate diagnosis before the age of 15. The usual presentation for the subjects in this study presenting in childhood was neonatal jaundice [7]. These episodes can occur spontaneously but are often triggered by physiological stressors [8]. The underlying ADAMTS13 deficiency in congenital TTP can be addressed by treatment with fresh frozen plasma (FFP), which contains physiological amounts of the ADAMTS13 enzyme, thus prophylactic administration of FFP can be an effective patient management strategy for this condition. To this end, Barbot et al. describe a case of a 14-year-old girl with intractable TTP that initially improved with FFP treatment, but consistently relapsed. She was therefore given prophylactic FFP infusions at a dose of 10 mg/kg once every three weeks, which prevented further TTP relapse during the 10 years of follow-up reported in this case [9].

## Case presentation

**Figure 1:**
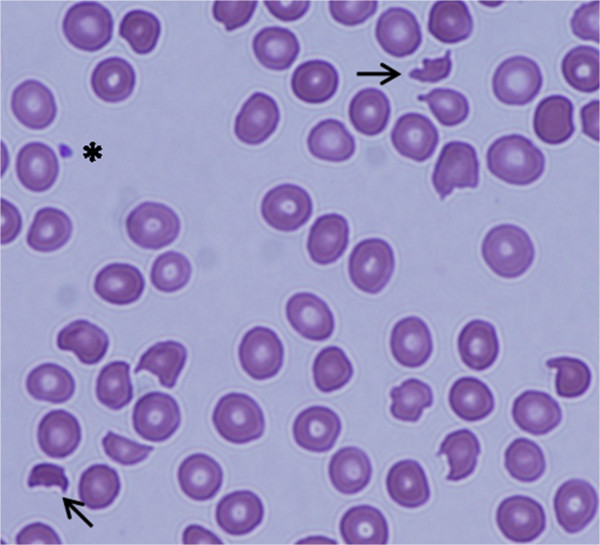
**Peripheral blood film showing schistocytes and marked decrease in platelet number indicating microangiopathic hemolytic anemia.**

We present a case of a 25 year old Caucasian woman with no know family history of hematological disorders with a history of thrombocytopenia since childhood who was ultimately diagnosed with and treated for congenital TTP at the age of 25. She presented at the age of 22 with a platelet count of 20 × 10^9^/L, an elevated lactate dehydrogenase (LDH) level of 473 U/L (normal range: 98-192 U/L), and an indirect bilirubin of 57.0 umol/L (normal range: 0.0-12.0 umol/L). She was completely asymptomatic, though she described a history of recurrent episodic easy bruising, jaundice, diarrhea, and fevers since infancy. As a child she also experienced episodes of epistaxis, gingival bleeding, and hematuria. She is Caucasian with no personal or family history of hematological disorders or consanguinity, and no family members known to share her clinical presentation. She was initially diagnosed with chronic immune thrombocytopenic purpura (ITP) and Evans syndrome as a child, and received various treatment regimens including IVIG as well as numerous courses of prednisone and cyclosporine, none of which significantly improved her condition. Treatment options were discussed and since she was asymptomatic and her platelet levels were felt to be safe the patient elected to proceed with expectant therapy and regular follow-up, which was relatively uneventful for several years. At the age of 25 she presented to another medical institution with fever, chills, vomiting, and dark urine. Blood work was significant for elevated creatinine, a platelet count of 10 × 10^9^/L, hemoglobin of 94 g/L and schistocytes in the peripheral smear. She was admitted to hospital with a diagnosis of TTP. Her attending physician ordered ADAMTS13 testing on the suspicion of congenital TTP. The ADAMTS13 functional assay showed an activity level of less than 10% and there was no evidence of anti-ADAMTS13 antibodies, establishing the diagnosis of congenital TTP. She ultimately recovered without undergoing plasma infusions and was discharged home. Regular screening blood work continued to show evidence of mild hemolysis. Five months after discharge the patient’s blood work showed schistocytes in the peripheral smear and a platelet count of 20 × 10^9^ (Figure 1). Her blood work was also significant for indirect bilirubin of 42.9 umol/L, and a LDH level of 491 U/L. She was admitted to our inpatient service and treated with a plasma infusion at 60 ml/h continuously for a 24 hour period, which resulted in a marked improvement in her thrombocytopenia and hemolysis. One month after being discharged from hospital she again developed marked thrombocytopenia with a platelet count of 19 × 10^9^/L and laboratory indications of hemolysis. She was admitted to hospital, treated successfully, and discharged home with a plan to start prophylactic plasma infusions at a dose of 10 mg/kg every three weeks. Unfortunately, three weeks later and just prior to receiving her first prophylactic plasma infusion, she relapsed and was again admitted for treatment.

Given her recurrent episodes of TTP, the patient was scheduled to receive weekly prophylactic plasma infusions at a dose of 10 mg/kg. This regimen was followed for five weeks before the treatment interval was increased. At the time of preparation of this manuscript our patient is maintained on biweekly plasma infusions; she has not developed any further episodes of acute hemolysis and her platelet count remains normal.

## Conclusion

Congenital TTP is a rare disorder caused by inherited ADAMTS13 deficiency, presenting as recurrent episodes of MAHA. Our patient is an interesting case from a clinicopathological perspective since, despite a long history of thrombocytopenia and hemolysis; however during these earlier episodes there was no evidence of end organ damage. She did not present with clear evidence of microangiopathy with schistocytes and elevated creatinine until the age of 25. Though rare, congenital TTP should be included in any differential diagnosis that includes acquired TTP, especially in the context of intractable TTP. Our case illustrates the importance of a proper diagnosis of this condition, which warrants long term follow-up and may require prophylactic FFP infusions. The decision to initiate prophylactic plasma infusions for patients with congenital TTP depends on the severity and frequency of episodes of TTP. Prior to receiving a definitive diagnosis of congenital TTP, our patient had not experienced any episodes of TTP for several years; however, between remote episodes of TTP her laboratory parameters showed evidence of mild hemolysis and low platelet counts. This baseline hemolysis and thrombocytopenia, coupled with three consecutive relapsing episodes of TTP post-therapeutic FFP infusions, guided our decision to continue with regular prophylactic FFP. This decision was not made lightly, due to the inherent risks associated with administration of any blood product. To this end we keenly await the availability of the recombinant ADAMTS13 enzyme product for a potentially safer alternative to FFP for treatment of congenital TTP.

## Consent

Written informed consent was obtained from the patient for publication of this Case report and any accompanying images. A copy of the written consent is available for review by the Editor of this journal.

## Abbreviations

ADAMTS13: A Disintegrin And Metalloproteinase with Thrombospondin Motifs 13
TTPthrombotic: thrombocytopenic purpura.
